# Supplementary material for: Spontaneously established reverse electric field to enhance the performance of triboelectric nanogenerators via improving Coulombic efficiency
Source: Nat Commun. 2024 May 16;15:4167. doi: 10.1038/s41467-024-48456-1 (PMC11099027; doi:10.1038/s41467-024-48456-1)
Supplement: Supplementary file 1 — Supplementary information [file 41467_2024_48456_MOESM1_ESM.pdf]

Supplementary Information

Spontaneously established reverse electric field to enhance the performance of triboelectric nanogenerators via improving Coulombic efficiency

Yikui Gao[1,2], Lixia He[1,2], Di Liu[1,2,*], Jiayue Zhang[3], Linglin Zhou[1,2], Zhong Lin Wang[1,2,4,5,*], Jie Wang[1,2,*]

[1]Beijing Institute of Nanoenergy and Nanosystems, Chinese Academy of Sciences, Beijing 101400, P.R. China.

[2]School of Nanoscience and Technology, University of Chinese Academy of Sciences, Beijing 100049, P.R. China.

[3]Department of Mechanical Engineering, Tsinghua University, Beijing 100084, P. R. China.

[4]Georgia Institute of Technology, Atlanta, 30332, USA.

[5]Yonsei Frontier Lab, Yonsei University, Seoul 03722, Republic of Korea.

*Corresponding Authors:

D. Liu: liudi@binn.cas.cn;

Z. L. Wang: zhong.wang@mse.gatech.edu;

J. Wang: wangjie@binn.cas.cn

Content

Supplementary Figures:

Supplementary Figure 1. The power management of TENG.

Supplementary Figure 2. The output charge of DC-TENG when the potential at node A increases.

Supplementary Figure 3. The actual limitations of CMEO for quantifying the performance of TENGs

Supplementary Figure 4. The test method for charge density at insulator's side surface

Supplementary Figure 5. The residual charge density on the side surface of insulator without external charge supplementation

Supplementary Figure 6. The simulated electric field around the electrode edge

Supplementary Figure 7. The self-regulation property of SEREF

Supplementary Figure 8. The detail mechanism for DC-TENG.

Supplementary Figure 9. Experimental verification of the principle of suppressing electrostatic breakdown of pasting an insulator at the edge of FE in DC-TENG.

Supplementary Figure 10. The cross section of the DC-TENG

Supplementary Figure 11. The observation experiments for corona discharge

Supplementary Figure 12. The output voltage of DC-TENG when the C_{test} is different.

Supplementary Figure 13. The breakdown threshold of 3rd BD.

Supplementary Figure 14. The reliability of the DC-TENG

Supplementary Figure 15. Q_{SC}, I_{SC} and V_{OC} of DC-TENG with different structure parameters

Supplementary Figure 16. The output power of DC-TENG with different structure parameters

Supplementary Figure 17. The electric field simulated result at FE edge with different gap between CCE and FE

Supplementary Figure 18. The testing method for output energy of TENG.

Supplementary Figure 19. The output energy of DC-TENG.

Supplementary Figure 20. The output energy of TENG calculated by ideal CMEO

Supplementary Figure 21. The curve for real energy output of TENG

Supplementary Figure 22. The ideal *Q*-*V* curve of DC-TENG obtained from CREO

Supplementary Figure 23. The maximum output energy calculated by Coulombic efficiency

Supplementary Figure 24. The calculation process of DC-TENG's output voltage

Supplementary Figure 25. The *Q*-*V* curve of DC-TENGs with different structure parameters obtained by linearly fitting the experimental data

Supplementary Figure 26. The maximum output energy comparation of DC-TENG.

Supplementary Figure 27. The schematic diagram and *Q*-*V* curve of CDC-TENG with different structure parameters

Supplementary Figure 28. The schematic diagram and *Q*-*V* curve of DEDC-TENG with different structure parameters

Supplementary Figure 29. The structure schematic diagram and power of rotary-mode DC-TENG (K = 3)

Supplementary Figure 30. The calculation process of rotary mode DC-TENG's output voltage

Supplementary Figure 31. The detailed structural diagram of the rotary mode DC-TENG (K = 9)

Supplementary Figure 32. Comparison of *Q*-*V* curves between MDC-TENG (K = 100) and DC-TENG with insulator (K = 9**)**

Supplementary Figure 33. The schematic diagram and *Q*-*V* curve of AC-TENG with different structure parameters

Supplementary Figure 34. The breakdown theory of CS-TENG

Supplementary Figure 35. The output charge of AC-TENG with/without insulator

Supplementary Figure 36. The simulated around the edge of electrode

Supplementary Tables:

Supplementary Table 1. The Coulombic efficiency of DC-TENG with different structure parameters

Supplementary Table 2. The Coulombic efficiency of CDC-TENG with different structure parameters

Supplementary Table 3. The Coulombic efficiency of DEDC-TENG with different structure parameters

Supplementary Table 4. The detailed parameters of different rotation mode DC-TENGs

Supplementary Table 5. The Coulombic efficiency of microstructure-designed DC-TENG (K = 100) and DC-TENG with insulator (K = 9)

Supplementary Table 6. The Coulombic efficiency of AC-TENG with different structure parameters

Supplementary Table 7. The Coulombic efficiency of AC-TENG of double dielectric layer

Supplementary Table 8. The detailed sliding motion parameters

Supplementary Table 9. The detailed simulation parameters

Supplementary Notes:

Supplementary Note 1. The charge loss in TENG resulted from power management

Supplementary Note 2. The actual limitations of CMEO for quantifying the performance of TENGs

Supplementary Note 3. AC-TENG and DC-TENG

Supplementary Note 4. The relationship between the three breakdown domains of DC-TENG

Supplementary Note 5. Definition and testing method for the open-circuit voltage of TENG

Supplementary Note 6. The testing method for output energy of TENG

Supplementary Note 7. Comparison output power and output energy of TENG

Supplementary Note 8. The maximum output energy of TENG according to CMEO

Supplementary Note 9. The CREO of TENG

Supplementary Note 10. The maximum output energy of TENG calculated by Q-V curve

Supplementary Note 11. The Coulomb efficiency of MDC-TENG

Supplementary Note 12. Breakdown theory

Supplementary Note 13. Modulating the electric field intensity in the breakdown domain

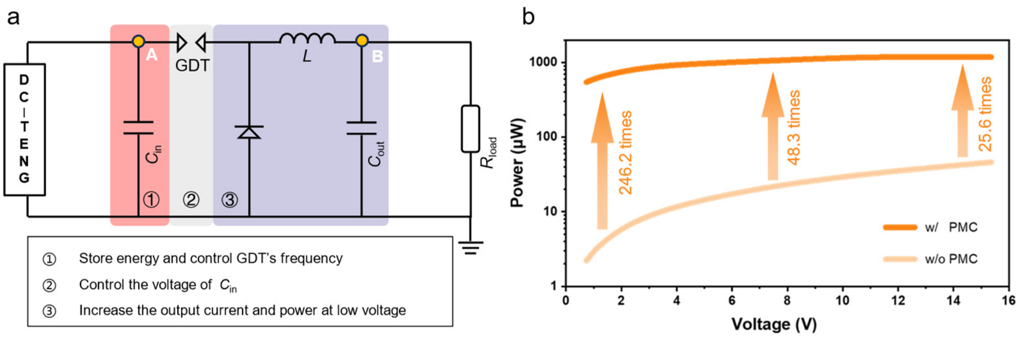

Supplementary Figure 1. The power management of TENG. (a) The represented power management circuit. **(b)** The output power of DC-TENG with different output voltage with/without power management circuit. The DC-TENG used in this experiment is shown in **Fig. 4h**. C_{in} is 55 pF, which is obtained by connecting four 220 pF capacitors in series. The threshold voltage of gas discharge tube (GDT) is 1500 V. The reverse breakdown voltage of freewheeling diode is 2000 V. L is 330 μH. C_{out} is 10 μF. Source data are provided as a Source data file.

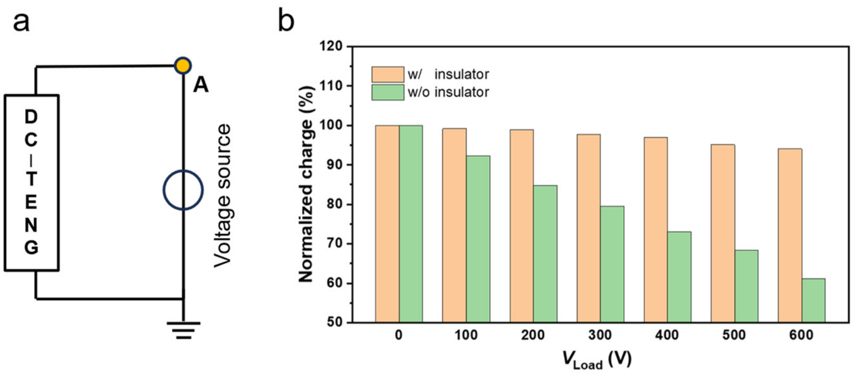

Supplementary Figure 2. The output charge of DC-TENG when the potential at node A increases. (a) The test circuit. The potential at node A can be controlled by changing the output voltage of the voltage source. **(b)** The output charge of DC-TENG. The DC-TENG used in this experiment is shown in **Fig. 2j and 2k**. Source data are provided as a Source data file.

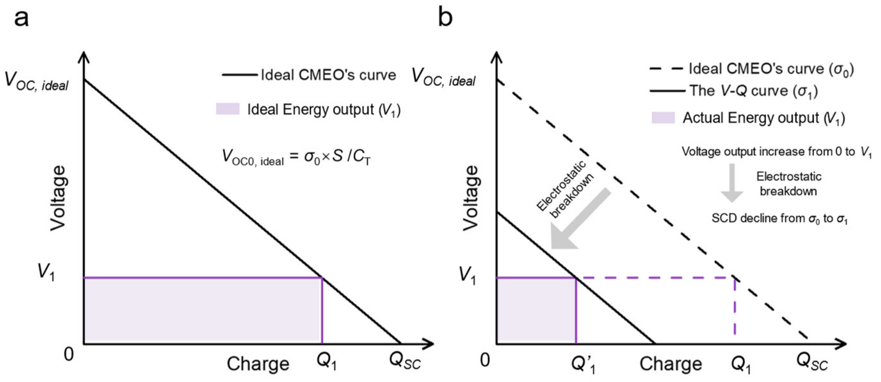

Supplementary Figure 3. The actual limitations of CMEO for quantifying the performance of TENGs. (a) The ideal energy output calculated by ideal CMEO's curve. (b) The actual energy output calculated by the actual *V-Q* curve (σ_1).

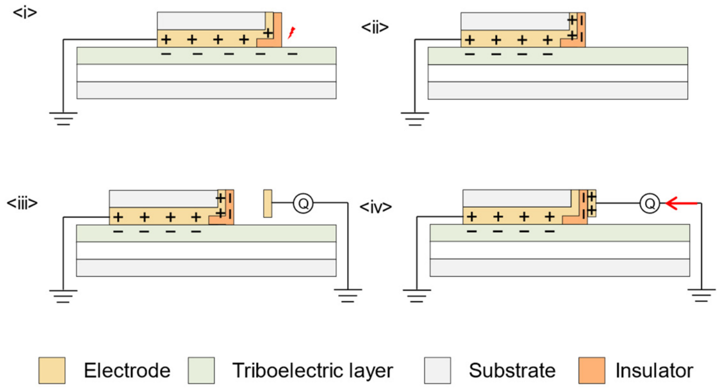

Supplementary Figure 4. The test method for charge density at insulator's side surface. <i> Negative charges are generated on the surface of triboelectric layer due to triboelectrification. A strong electric field will be generated at the edge of the electrode, causing electrostatic breakdown. <ii> Driven by Coulomb force, negative ions rapidly accumulate on the surface of insulator. <iii> The other metal electrode is connected to the charge meter and gradually approaches the insulator. <iv> When the electrode contacted with the insulator, positive charges will be induced in the insulator. The number of positive charges induced by the electrode is the same as the number of negative charges on the surface of insulator.

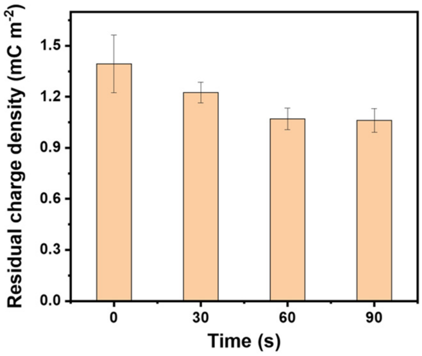

Supplementary Figure 5. The residual charge density on the side surface of insulator without external charge supplementation. After 30 s, 60 s and 90 s, the surface charge retention rate is 87.9%, 76.8% and 76.2%, respectively. The testing method for charge density on the side surface of insulator is shown in **Supplementary Fig. 4**. Error bars represent standard deviation with five trials. Source data are provided as a Source data file.

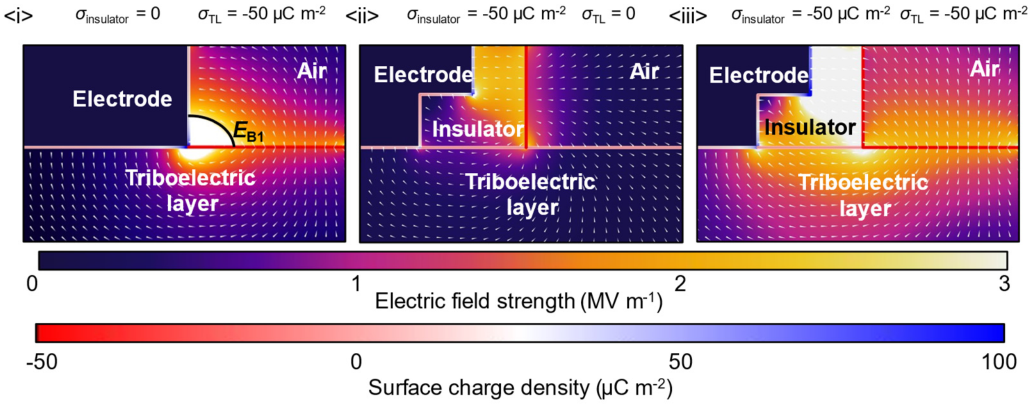

Supplementary Figure 6. The simulated electric field around the electrode edge. <i> The SCD of insulator ($\sigma_{\text{insulator}}$) is zero and the SCD of triboelectric layer (σ_{TL}) is -50 μC m^{-2}. The electric field strength is over E_{B1}. <ii> The SCD of insulator is -50 μC m^{-2} and the SCD of triboelectric layer is zero. It is worth noting that the direction of the electric field at the edge of the electrode is reversed. <iii> The SCD of insulator is -50 μC m^{-2} and the SCD of triboelectric layer is -50 μC m^{-2}. The electric field strength is below of E_{B1}.

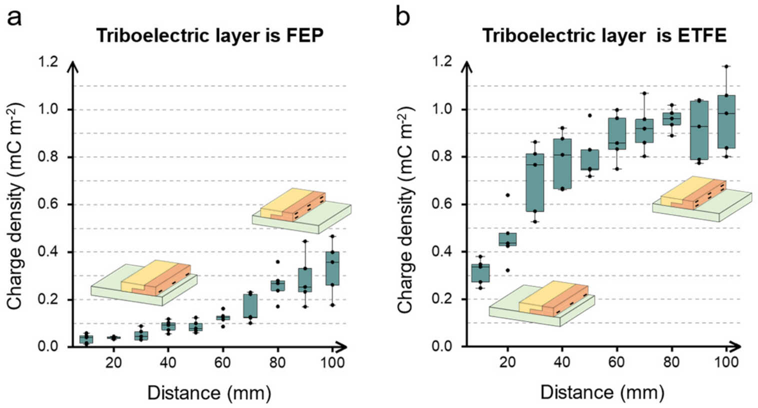

Supplementary Figure 7. The self-regulation property of SEREF. (a) When the triboelectric layer is Fluorinated ethylene propylene (FEP), the SCD of insulator's side surface rapidly accumulates and reaches to 0.3 mC m^{-2} when the moving distance is 10 cm. **(b)** When the triboelectric layer is Ethylene-terafluoroethlene (ETFE), the SCD of insulator's side surface rapidly accumulates and reaches to 1.0 mC m^{-2} when the moving distance is 10 cm. The accumulated charge density of insulator's side surface depends on the triboelectric process, and the enhanced triboelectric charge density will intensify the electrostatic breakdown and more charges will accumulate on the insulator surface, realizing the self-regulation behavior. The box plot illustrates the distribution of five sets of data, with the whiskers representing the most extreme values excluding outliers. Source data are provided as a Source data file.

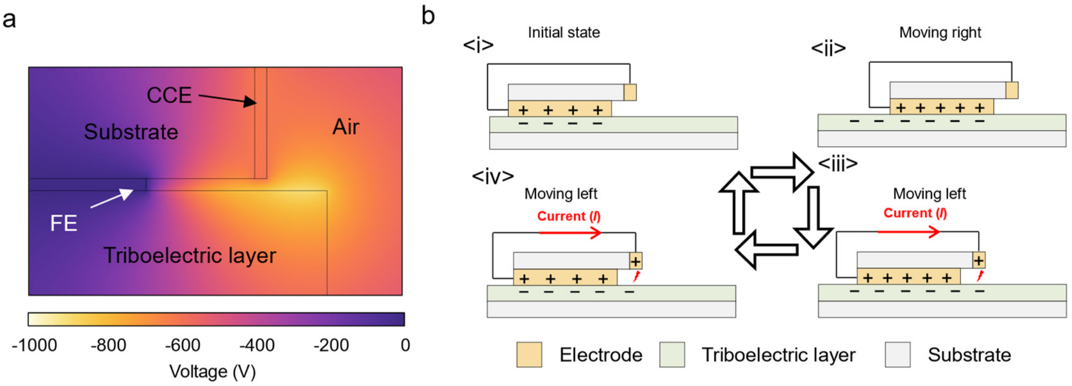

Supplementary Figure 8. The detailed mechanism for DC-TENG. (a) The simulated result of the potential difference between CCE and FE. Obviously, the potential of FE is higher than that of CCE, thus negative charges transfer from CCE to FE. (b) The detailed charge transfer mechanism for DC-TENG.

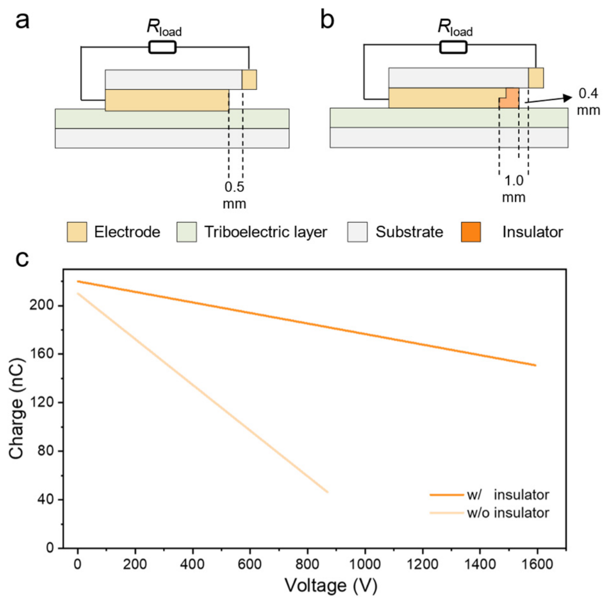

Supplementary Figure 9. Experimental verification of the principle of suppressing electrostatic breakdown of pasting an insulator at the edge of FE in DC-TENG. (a) The structure diagram of DC-TENG without insulator. The gap between CCE and FE is 0.5 mm. **(b)** The structure diagram of DC-TENG with insulator. There is still about 0.4 mm of air gap in the 2nd BD between CCE and FE. **(c)** The *Q-V* curve of DC-TENG. Previous work speculated that the insulator pasted at the edge of electrode was to completely replace the air in the 2nd BD, increasing the breakdown threshold of 2nd BD. Here, two experimental evidences indicate that it may be unreasonable. Firstly, when the insulator is nitrile, the effect of suppressing electrostatic breakdown is poor **(Fig. 4e-g)**. Furthermore, even in the presence of a minor air gap within the 2nd BD, the effect of suppressing air breakdown still exists. These can be explained by the theory proposed in this work. Source data are provided as a Source data file.

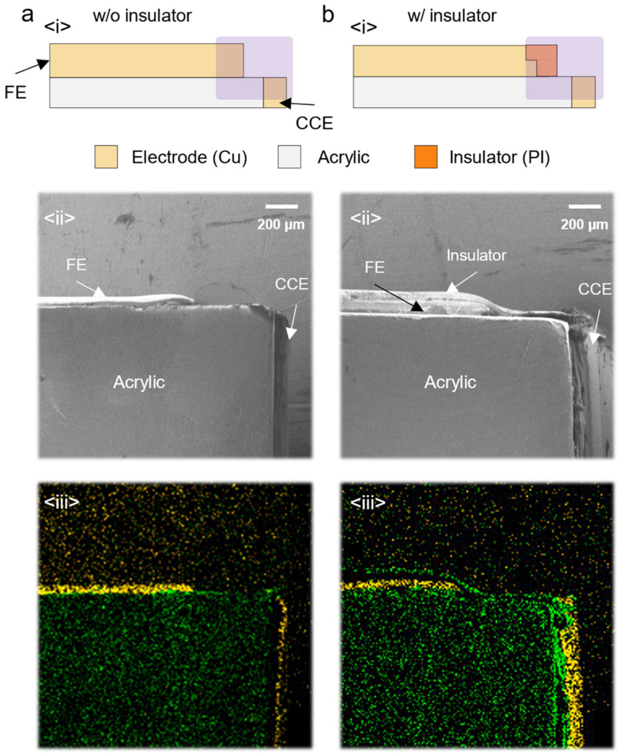

Supplementary Figure 10. The cross section of the DC-TENG. <i> The structure diagram of DC-TENG **(a)** without insulator, **(b)** with insulator. The purple shaded area is the observation area. <ii> SEM image of the observed area (scale bar: 200 μm). <iii> The distribution of elements at the observation area of DC-TENG was analyzed by using the energy-spectrum scanning function of SEM. The yellow dots represent copper element, green dots represent carbon element. Due to the devices are handmade and the cross section is relatively rough, it is difficult to distinguish the positional relationship between electrodes and insulator solely based on SEM images. Therefore, we have supplemented the element distribution at the observation area. Due to the substrate of the device is acrylic (Polymeric Methyl Methacrylate, PMMA) and the insulation layer is polyimide (PI), their main element is carbon and does not contain copper. The main element of the electrode is copper and does not contain carbon. Therefore, the electrodes and insulator can be distinguished by analyzing the distribution of carbon and copper elements at the observation area.

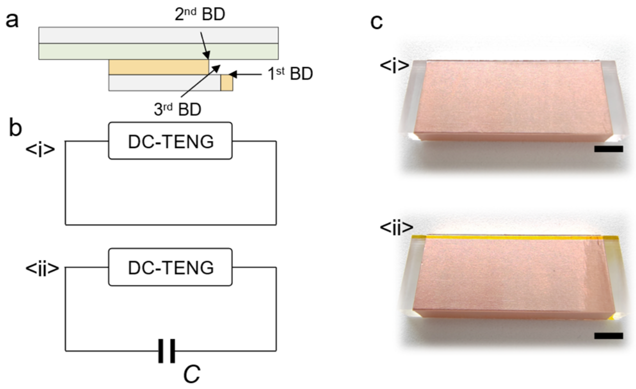

Supplementary Figure 11. The observation experiments for corona discharge. (a) Structural schematic diagram for observation experiments. Here, the slider is the TL, and the other part composed of FE and CCE is as the stator. To realize convenient observation, transparent ETFE is used as TL. **(b)** <i> The equivalent circuit of observing corona discharge in 1st BD and 2nd BD. <ii> The equivalent circuit of observing spark discharge in 3rd BD. The capacitor is used to increase $V_{CCE\text{-}FE}$ to the voltage threshold of 3rd BD. **(c)** <i> Physical image of DC-TENG w/o insulator (Scale bar: 5 mm). <ii> Physical image of DC-TENG w/ insulator (Scale bar: 5 mm).

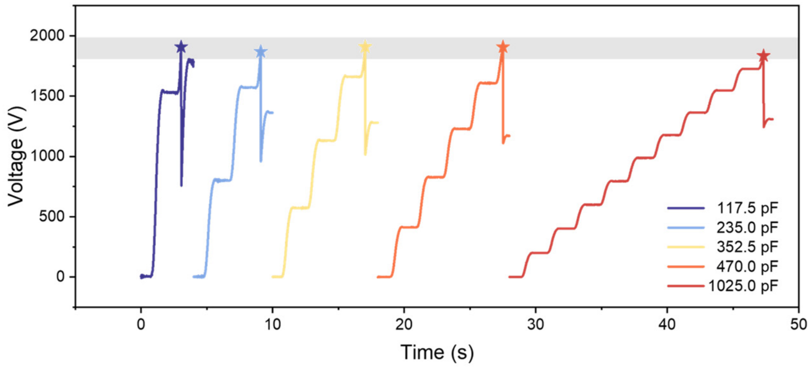

Supplementary Figure 12. The output voltage of DC-TENG when the C_{test} is different. The experimental results indicate that C_{test} has no effect on the breakdown of the 3rd BD of DC-TENG. Source data are provided as a Source data file.

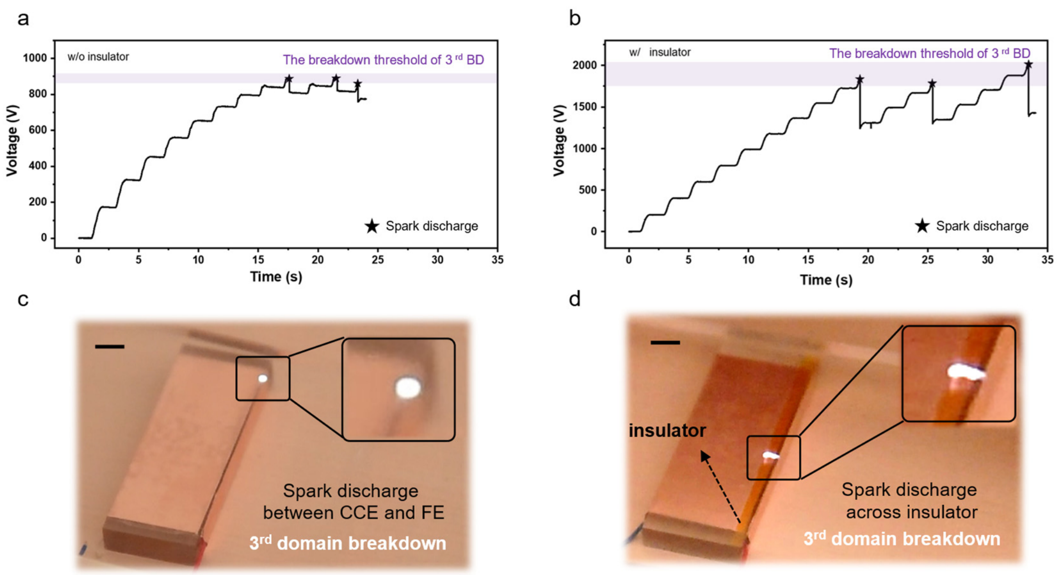

Supplementary Figure 13. The breakdown threshold of 3rd BD. (a) The output voltage of DC-TENG without insulator. **(b)** The output voltage of DC-TENG with insulator. The output voltage of DC-TENG will no longer over the certain value due to the occurrence of 3rd BD. **(c)** and **(d)** are the photos of spark discharge of 3rd BD (Scale bar: 5 mm). Source data are provided as a Source data file.

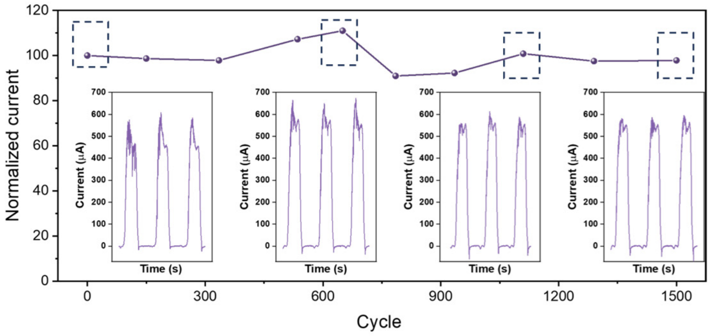

Supplementary Figure 14. The reliability of the DC-TENG. Source data are provided as a Source data file.

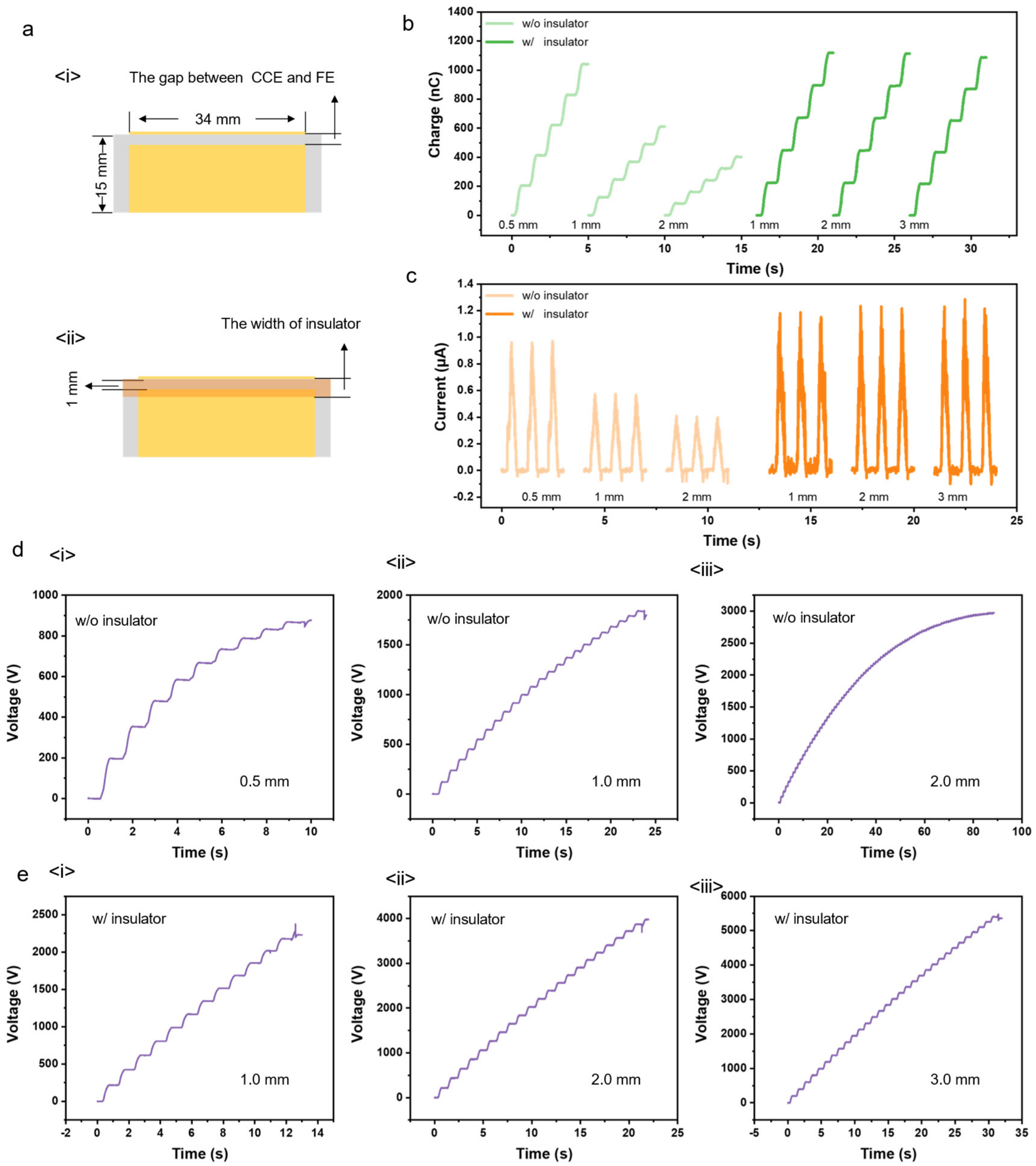

Supplementary Figure 15. Q_{SC}, I_{SC} and V_{OC} of DC-TENG with different structure parameters. (a) <i> The structure parameters of DC-TENG w/o insulator. The gap between CCE and FE is 0.5 mm, 1.0 mm and 2.0 mm, respectively. <ii> The structure parameters of DC-TENG w/o insulator. The width of insulator is 1.0 mm, 2.0 mm and 3.0 mm, respectively. **(b)** Q_{SC} of DC-TENG. **(c)** I_{SC} of DC-TENG. **(d)** V_{OC} of DC-TENG w/o the insulator. **(e)** V_{OC} of DC-TENG w/ insulator. Source data are provided as a Source data file.

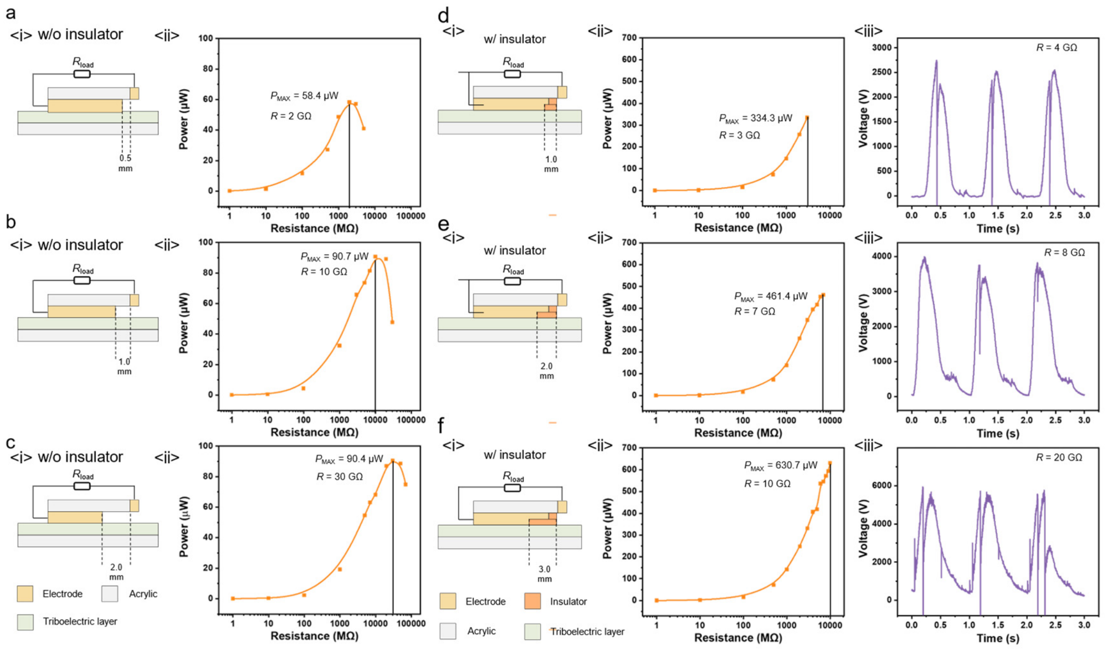

Supplementary Figure 16. The output power of DC-TENG with different structure parameters. (a) <i> The structure diagram of DC-TENG without insulator. **<ii>** The output power of DC-TENG. The gap between CCE and FE is 0.5 mm. **(b) <i>** The structure diagram of DC-TENG without insulator. **<ii>** The output power of DC-TENG. The gap between CCE and FE is 1.0 mm. **(c) <i>** The structure diagram of DC-TENG without insulator. **<ii>** The output power of DC-TENG. The gap between CCE and FE is 2.0 mm. **(d) <i>** The structure diagram of DC-TENG with insulator. The width of insulator is 1.0 mm.**<ii>** The output power and **<iii>** the output voltage (The resistance is 4 GΩ.) of DC-TENG. **(e) <i>** The structure diagram of DC-TENG with insulator. The width of insulator is 2.0 mm.**<ii>** The output power and **<iii>** the output voltage (The resistance is 7 GΩ.) of DC-TENG. **(f) <i>** The structure diagram of DC-TENG with insulator. The width of insulator is 3.0 mm.**<ii>** The output power and **<iii>** the output voltage (The resistance is 20 GΩ.) of DC-TENG. Obviously, when a breakdown signal occurs in the current signal, the corresponding output voltage approaches the threshold voltage of the 3rd BD (i.e. the open circuit voltage of DC-TENG). Source data are provided as a Source data file.

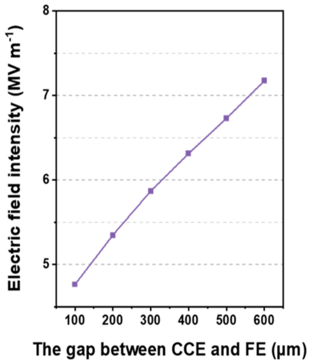

Supplementary Figure 17. The electric field simulated result at FE edge with different gap between CCE and FE. Source data are provided as a Source data file.

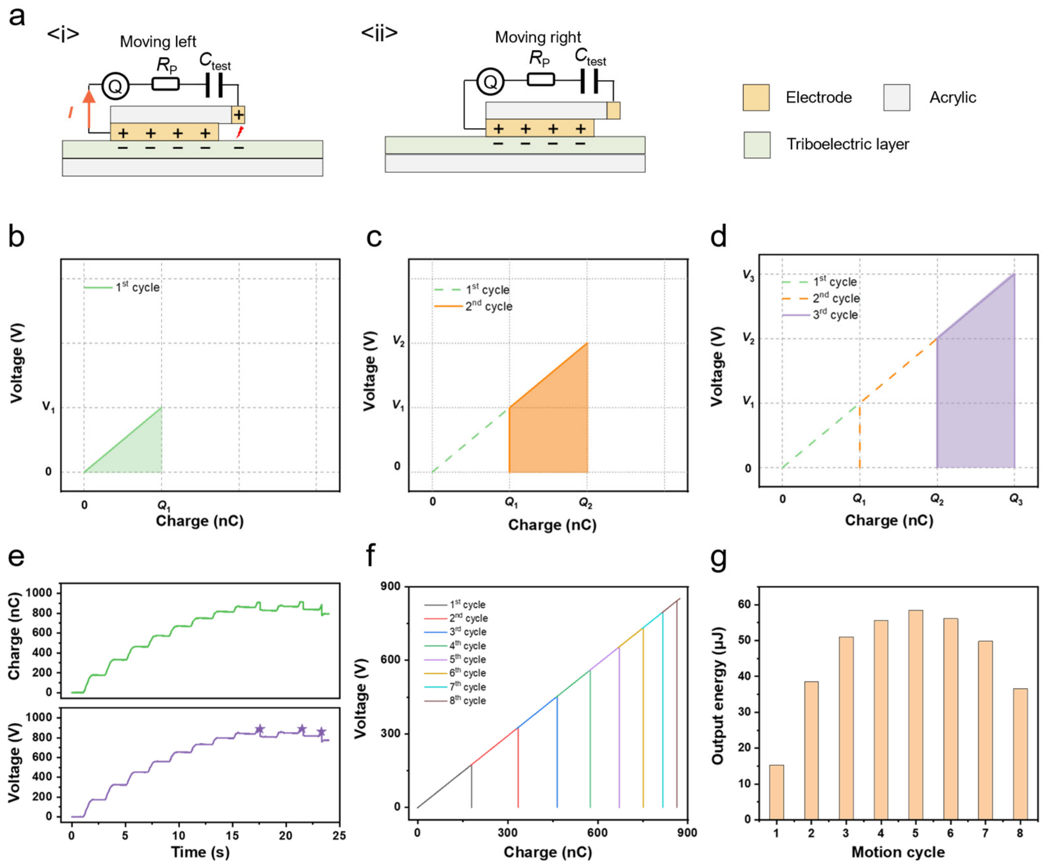

Supplementary Figure 18. The testing method for output energy of TENG. (a) The schematic diagram of the testing circuit. **<i>** When the DC-TENG moves left, current flows from FE to CCE and charge is stored in the testing capacitor (C_{test}). **<ii>** When the DC-TENG moves right, current is zero. Rp is a protective resistor, which is utilized to protect the charge meter. **(b)-(d)** The schematic diagram of testing output energy for DC-TENG. The green/orange/purple shadow area represent the output energy of DC-TENG in the 1st/2nd/3rd motion cycle. Q_n is the total output charge after the nth motion cycle. Q_n-Q_{n-1} is the output charge of the nth motion cycle. V_n is the output voltage after the nth motion cycle. **(e)** The output charge and output voltage of DC-TENG (Without insulator, the gap between CCE and FE is 0.5 mm.). **(f)-(g)** The output energy of DC-TENG in the nth motion cycle (Without insulator, the gap between CCE and FE is 0.5 mm.). Source data are provided as a Source data file.

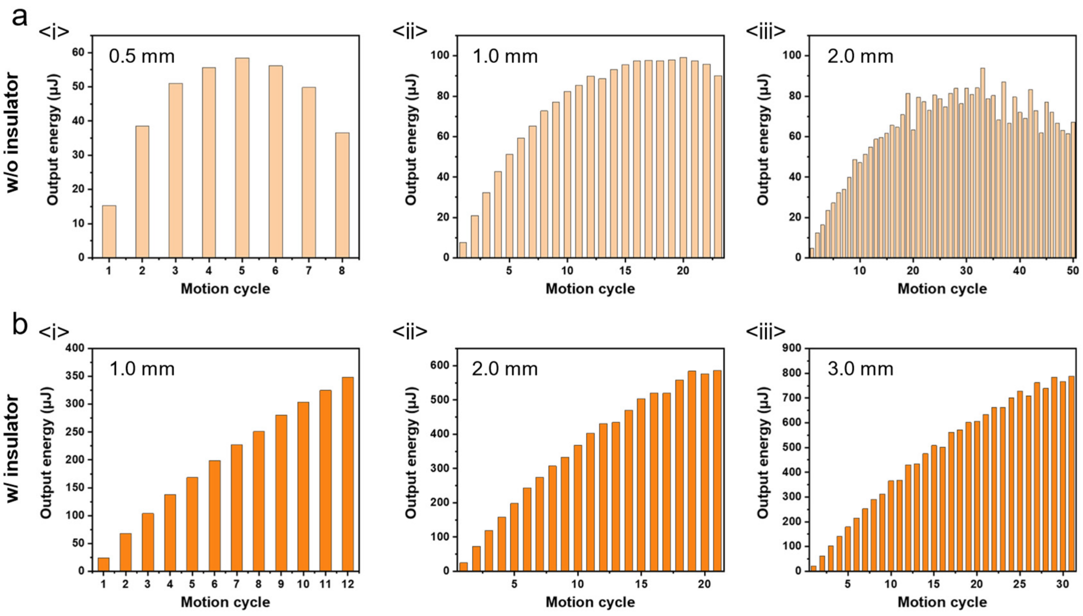

Supplementary Figure 19. The output energy of DC-TENG. (a) The output energy of DC-TENG without insulator. <i>-<iii> The gap between FE and CCE is 0.5 mm, 1.0 mm and 2.0 mm, respectively. The detailed structure diagram is shown in **Supplementary Fig. 16a-c**. **(b)** The output energy of DC-TENG with insulator. <i>-<iii> The width of insulator is 1.0 mm, 2.0 mm and 3.0 mm, respectively. The detailed structure diagram is shown in **Supplementary Fig. 16d-f**. Source data are provided as a Source data file.

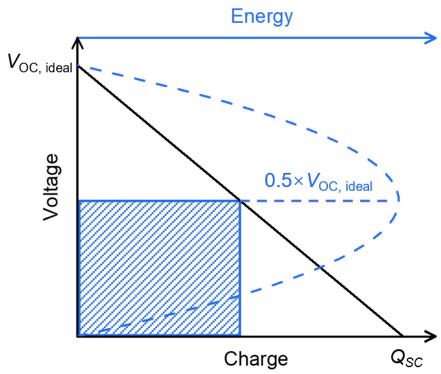

Supplementary Figure 20. The output energy of TENG calculated by ideal CMEO. The dashed line is the energy, and the solid line is the CMEO.

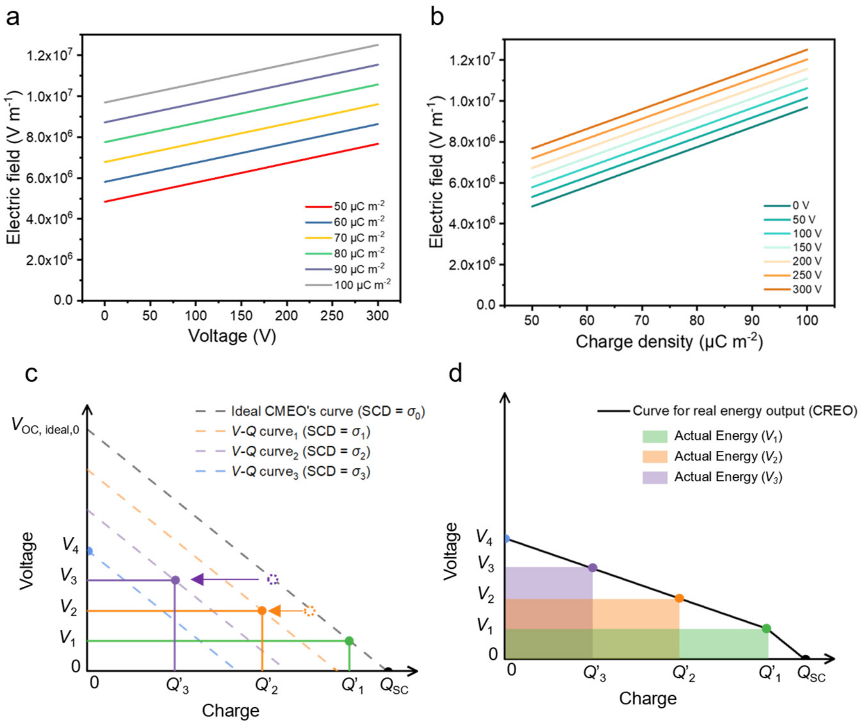

Supplementary Figure 21. The curve for real energy output of TENG. The relationship between **(a)** output voltage and **(b)** surface charge density with the electric field strength around electrode edge. **(c)** and **(d)** The curve for real energy output (CREO, the fold line). Q'_1, Q'_2 and Q'_3 are the actual output charge when the output voltage of TENG is V_1, V_2 and V_3, respectively. Source data are provided as a Source data file.

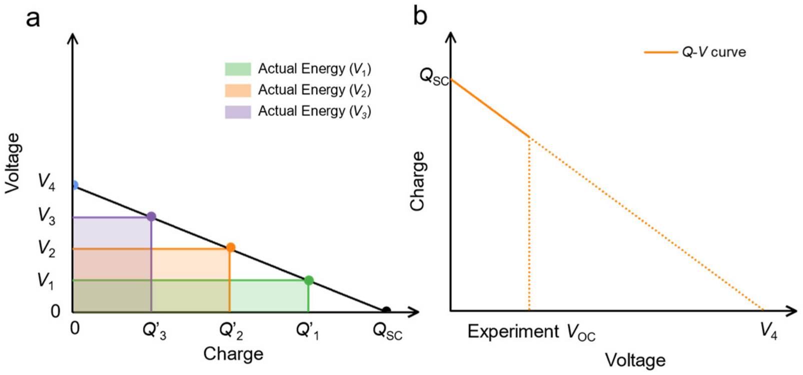

Supplementary Figure 22. The ideal *Q-V* curve of DC-TENG obtained from CREO. (a) The CREO (the straight line) of DC-TENG. (b) The ideal *Q-V* curve of DC-TENG obtained from CREO (voltage as the horizontal axis, charge as the vertical axis). Due to the maximum experiment output voltage (experiment V_{OC}) is lower than ideal open-circuit voltage ($V_{OC,\ ideal}$), thus the actual *Q-V* curve is shown as **Fig. 3g**.

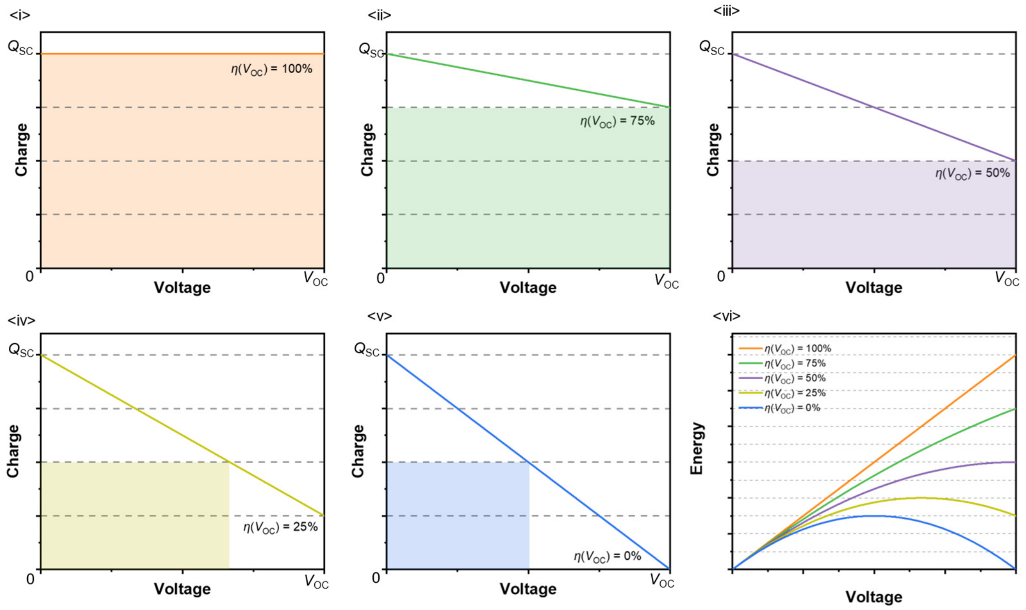

Supplementary Figure 23. The maximum output energy calculated by Coulombic efficiency. <i>-<v> *Q-V* curves of DC-TENGs, when their V_{OC} and Q_{SC} are same and their Coulomb efficiency are different. <vi> The output energy of DC-TENGs with same V_{OC} and Q_{SC}, and different Coulomb efficiency.

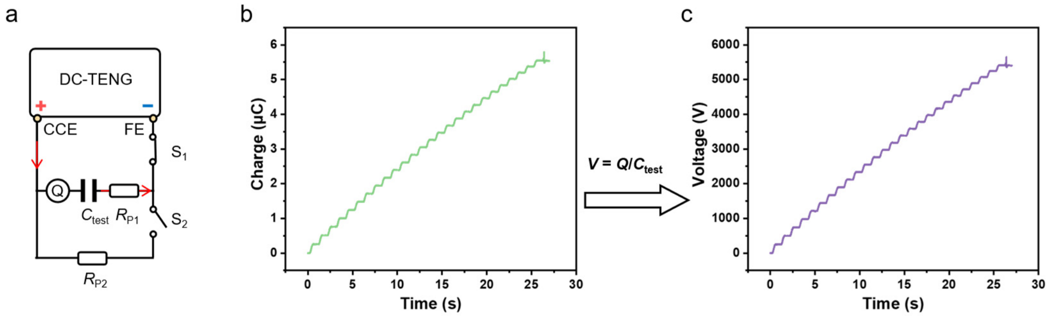

Supplementary Figure 24. The calculation process of DC-TENG's output voltage. **(a)** The testing circuit. **(b)** The total output charge of DC-TENG. **(c)** The output voltage of DC-TENG. Source data are provided as a Source data file.

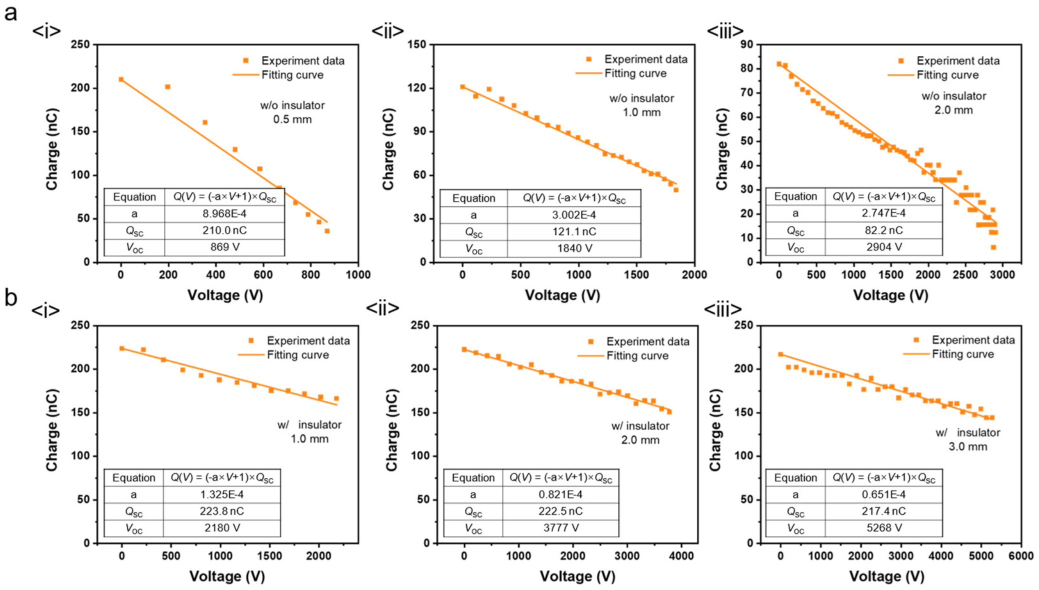

Supplementary Figure 25. The *Q-V* curve of DC-TENGs with different structure parameters obtained by linearly fitting the experimental data. (a) The *Q-V* curve of DC-TENG without insulator. <i>-<iii> The gap between FE and CCE is 0.5 mm, 1.0 mm and 2.0 mm, respectively. The detailed structure diagram is shown in **Supplementary Fig. 16a-c**. **(b)** The *Q-V* curve of DC-TENG with insulator. <i>-<iii> The width of insulator is 1.0 mm, 2.0 mm and 3.0 mm, respectively. The detailed structure diagram is shown in **Supplementary Fig. 16d-f**. The Coulombic efficiency of these devices is shown in **Supplementary Table 1.** Source data are provided as a Source data file.

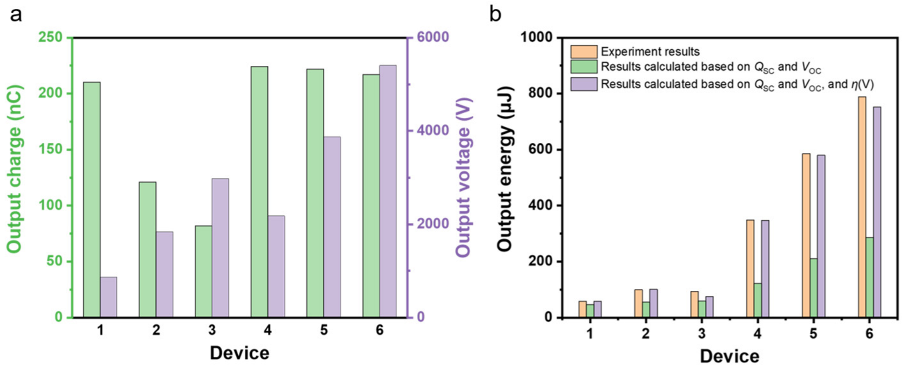

Supplementary Figure 26. The maximum output energy comparation of DC-TENG. (a) The output charge and output voltage of DC-TENG. **(b)** The output energy of DC-TENG. Source data are provided as a Source data file.

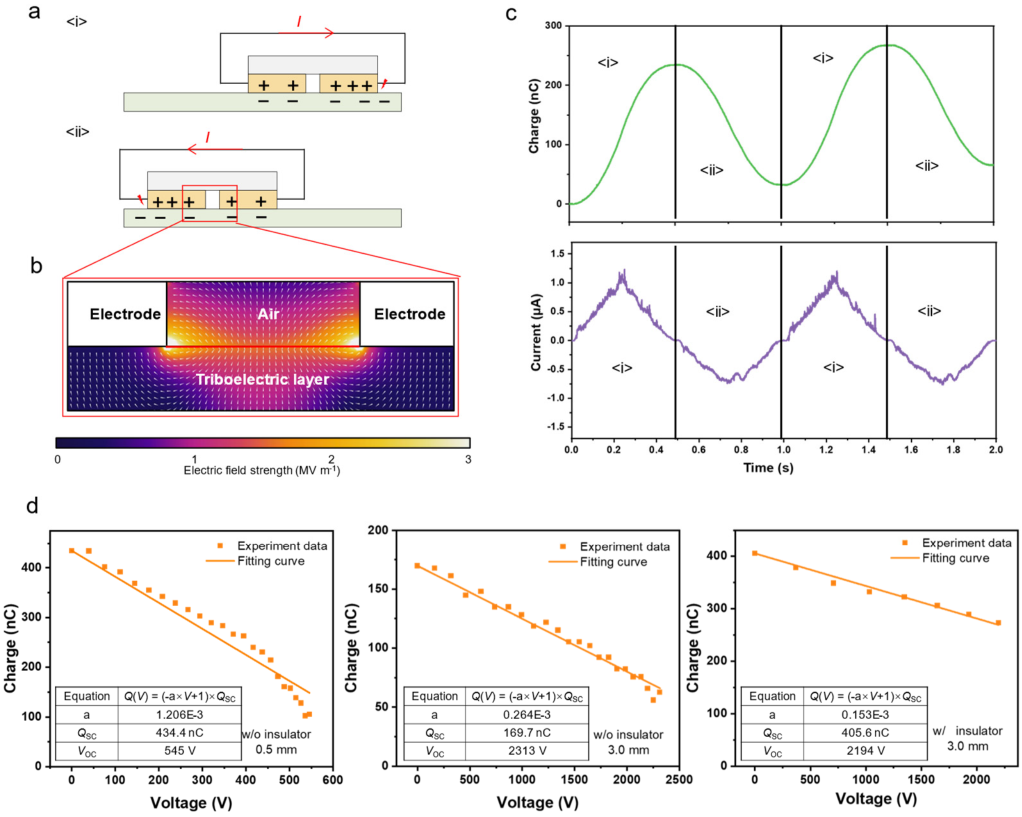

Supplementary Figure 27. The schematic diagram and *Q*-*V* curve of CDC-TENG with different structure parameters. (a) Schematic diagram of CDC-TENG. **(b)** The simulated electric field result of CDC-TENG. **(c)** Q_{SC} and I_{SC} of CDC-TENG. **(d)** The *Q*-*V* curve of CDC-TENG with different structure parameters obtained by linearly fitting the experimental data. Source data are provided as a Source data file.

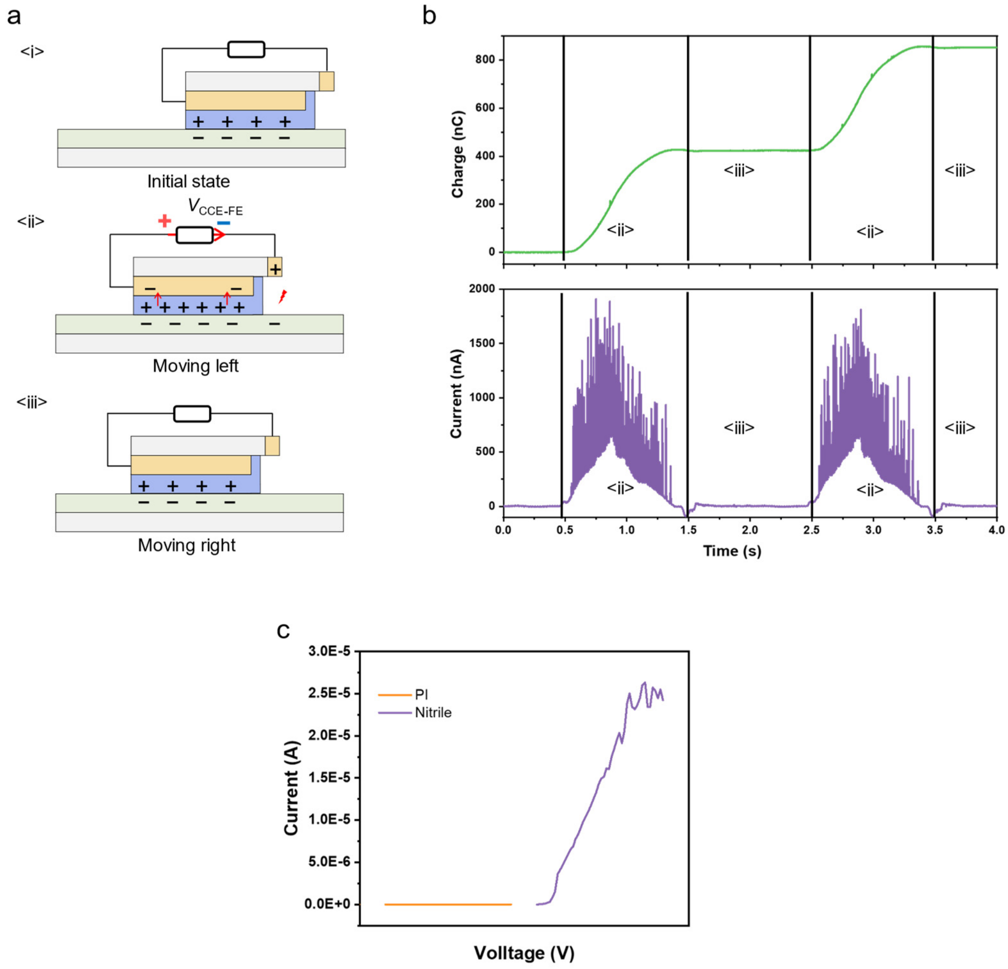

Supplementary Figure 28. The schematic diagram and *Q-V* curve of DEDC-TENG with different structure parameters. (a) Schematic diagram of DEDC-TENG. **(b)** Q_{SC} and I_{SC} of DEDC-TENG. **(c)** The leakage current of PI and nitrile (The testing voltage increases from 0 V to 500 V.). Source data are provided as a Source data file.

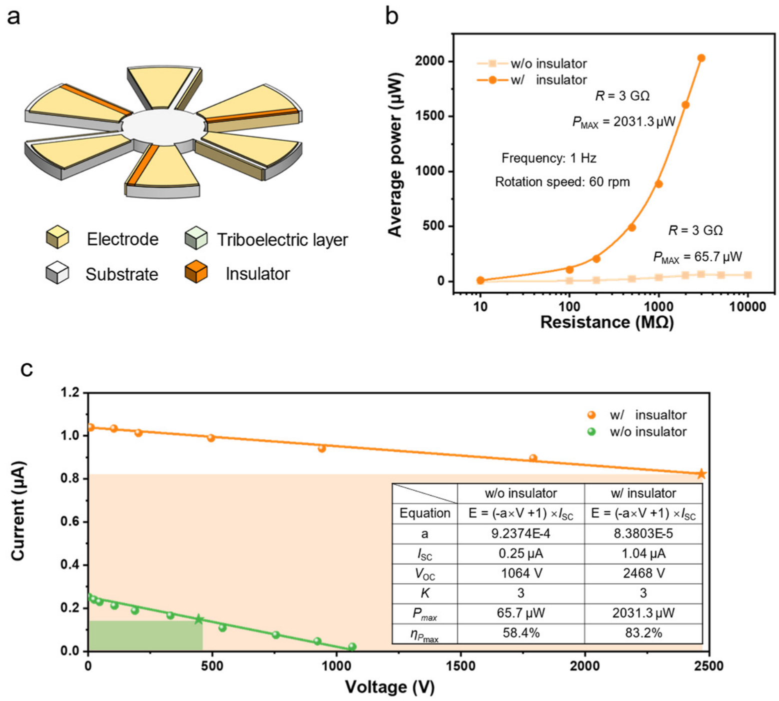

Supplementary Figure 29. The structure schematic diagram and power of rotary-mode DC-TENG (K = 3). (a) The structure schematic diagram of the rotary mode DC-TENG. To ensure consistent testing conditions, two sets of DC-TENG with different structures were fabricated in one device, each with three units. **(b)** The output power of the rotary mode DC-TENG. **(c)** The I-V curve of rotary-mode DC-TENG (K = 3). The shaded area represents the maximum power output. Source data are provided as a Source data file.

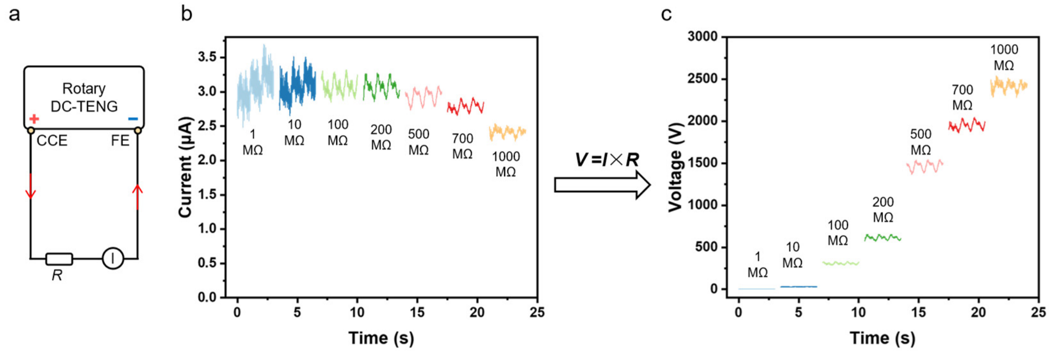

Supplementary Figure 30. The calculation process of rotary mode DC-TENG's output voltage. (a) The testing circuit. **(b)** The output current and of **(c)** output voltage the rotary mode DC-TENG (K = 9) with different resistance. Source data are provided as a Source data file.

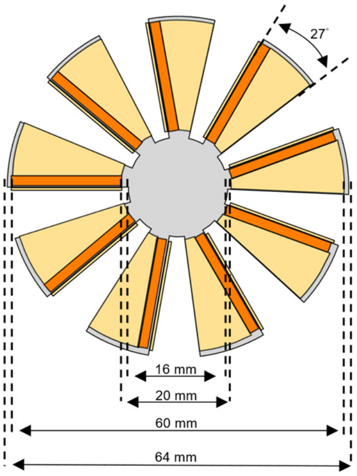

Supplementary Figure 31. The detailed structural diagram of the rotary mode DC-TENG (K = 9).

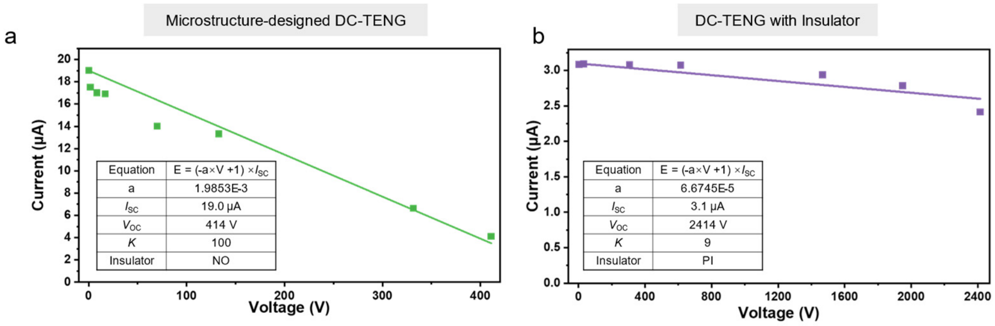

Supplementary Figure 32. Comparison of *Q-V* curves between MDC-TENG (K = 100) and DC-TENG with insulator (K = 9). (a) The *I-V* curve of microstructure-designed DC-TENG (K = 100). (**b**) The *I-V* curve of DC-TENG with insulator (K = 9). Source data are provided as a Source data file.

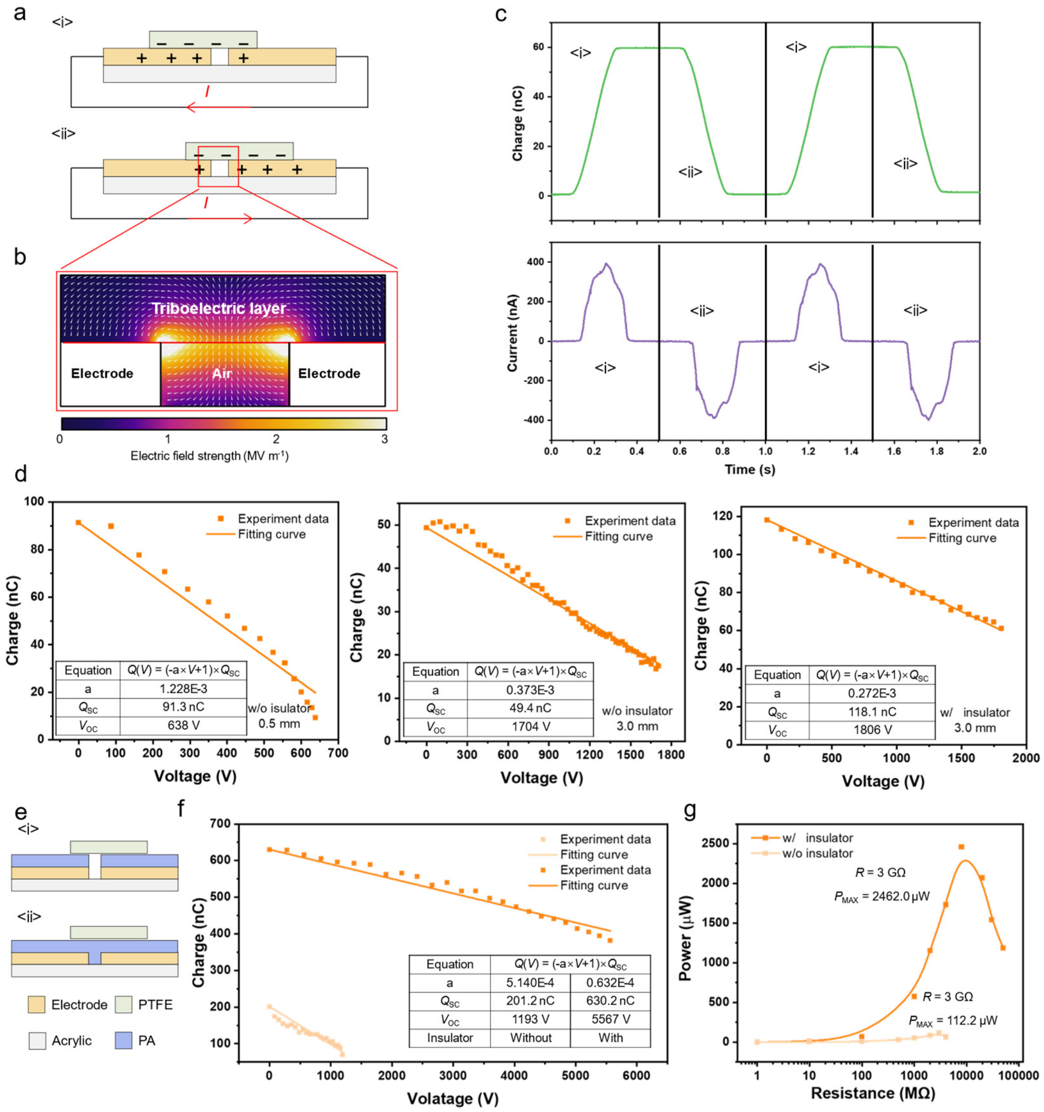

Supplementary Figure 33. The schematic diagram and *Q-V* curve of AC-TENG with different structure parameters. (a) Schematic diagram of AC-TENG. **(b)** The simulated electric field result of AC-TENG. **(c)** Q_{SC} and I_{SC} of AC-TENG. **(d)** *Q-V* curve of AC-TENG with different structure parameters obtained by linearly fitting the experimental data. **(e)** Schematic diagram of double dielectric layer (PTFE-PA) AC-TENG. <i> PA does not envelop the edge of the electrodes (w/o insulator); <ii> PA envelops the edge of the electrode (w/ insulator). **(f)** The *Q-V* curve obtained by linearly fitting the experimental data. **(g)** The output power of double dielectric layer AC-TENG. Source data are provided as a Source data file.

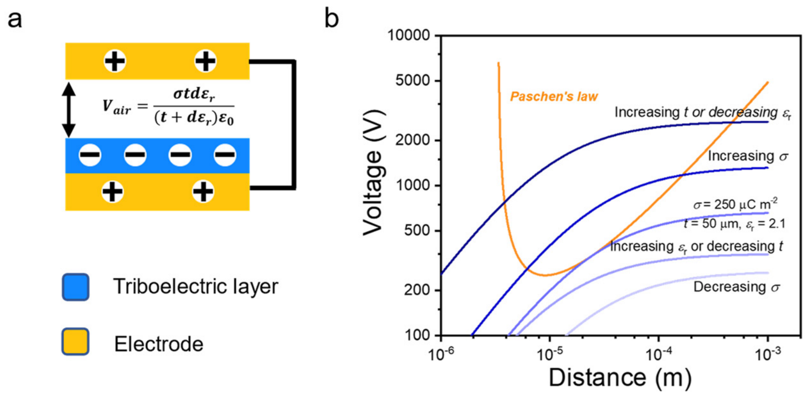

Supplementary Figure 34. The breakdown theory of CS-TENG. (a) The structure diagram of CS-TENG. V_{air} is the air gap voltage of CS-TENG. σ is the surface charge density of triboelectric layer. t and ε_r are the thickness and relative dielectric constant of triboelectric layer, respectively. ε_0 is 8.85×10^{-12} F m^{-1}. **(b)** The relationship between V_b curve (the orange line) and V_{air} (the blue lines). When σ is 250 μC m^{-2}, t is 50 μm, ε_r is 2.1, air breakdown does not occur due to two curves are tangent, and 250 μC m^{-2} is the maximum surface charge density. If increasing σ, increasing t or decreasing ε_r, then the two curves intersect and air breakdown occurs. If decreasing σ, decreasing t or increasing decreasing ε_r, then the two curves separate and air breakdown does not occur. Source data are provided as a Source data file.

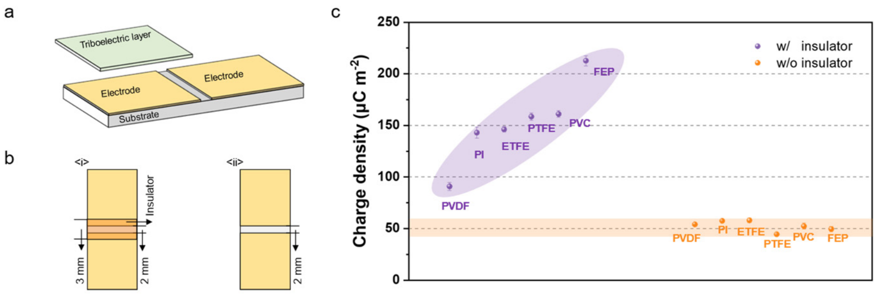

Supplementary Figure 35. The output charge of AC-TENG with/without insulator. (a) The structure diagram of AC-TENG. **(b)** <i> AC-TENG with insulator. <ii> AC-TENG without insulator. **(c)** The surface charge density of AC-TENG. Source data are provided as a Source data file.

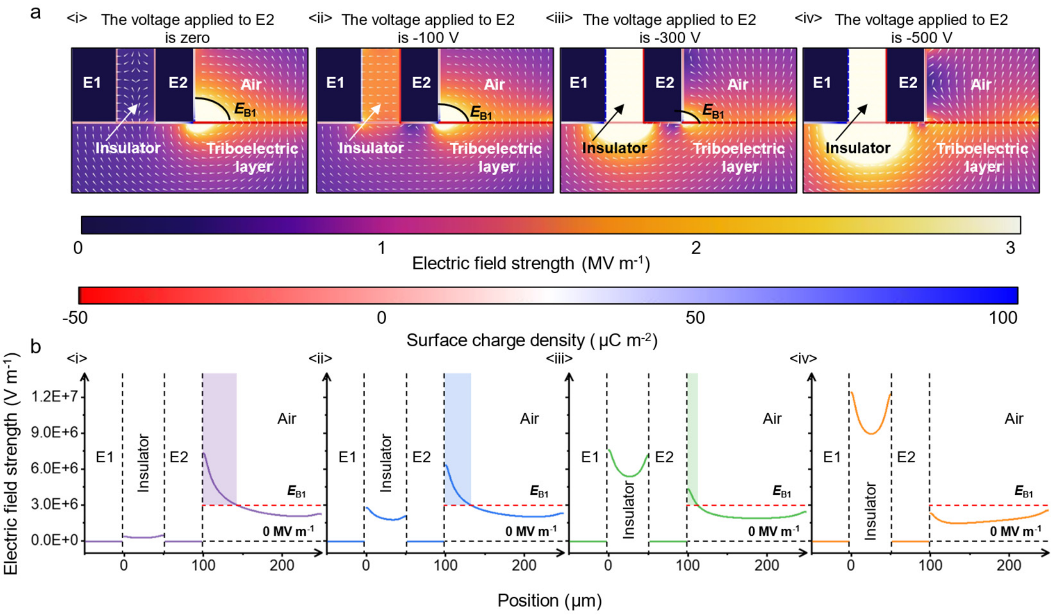

Supplementary Figure 36. The simulated electric field around the edge of electrode (surface charge density of the triboelectric layer is -50 μC m^{-2}.). E2 is used to modulate the electric field. The insulator between electrodes is mainly used for electrode isolation. <i>-<iv> As the voltage applied to E2 increases from zero to -500 V, the region of the breakdown domain gradually decreases. The data in **(b)** is taken from the electric field intensity at 5 μm above the triboelectric layer in **(a)**. Source data are provided as a Source data file.

Supplementary Table 1. The Coulombic efficiency of DC-TENG with different structure parameters

	a	Q_{SC}(nC)	V_{OC}(V)	*η*(500V)	*η*(869V)	*η*(1840V)	*η*(2180V)	*η*(2904V)	*η*(3777V)	*η*(5268V)
w/o insulator (0.5 mm)	8.968E-4	210.0	869	55.2%	**22.1%**	_	_	_	_	_
w/o insulator (1.0 mm)	3.002E-4	121.1	1840	85.0%	73.9%	**44.8%**	_	_	_	_
w/o insulator (2.0 mm)	2.747E-4	82.2	2904	86.3%	76.1%	49.4%	40.1%	**20.2%**	_	_
w/ insulator (1.0 mm)	1.325E-4	223.8	2180	93.4%	88.5%	75.6%	**71.1%**	_	_	_
w/ insulator (2.0 mm)	0.821E-4	222.5	3777	95.9%	92.9%	84.9%	82.1%	76.2%	**69.0%**	_
w/ insulator (3.0 mm)	0.651E-4	217.4	5268	96.7%	94.3%	90.2%	85.8%	81.1%	75.4%	**65.7%**

Supplementary Table 2. The Coulombic efficiency of CDC-TENG with different structure parameters

	Q_{SC}(nC)	V_{OC}(V)	*η*(500V)	*η*(1000V)	*η*(1500V)	*η*(2000V)
w/o insulator (0.5 mm)	434.4	545	39.7%	_	_	_
w/o insulator (3.0 mm)	169.7	2313	86.8%	73.6%	60.4%	47.2%
w/ insulator (3.0 mm)	405.6	2194	92.3%	84.7%	77.0%	69.4%

Supplementary Table 3. The Coulombic efficiency of DEDC-TENG with different structure parameters

	Q_{SC}(nC)	V_{OC}(V)	*η*(500V)	*η*(1000V)	*η*(1500V)	*η*(2000V)
DEDC-TENG (Nitrile)	359.0	2040	88.9%	77.8%	66.7%	55.6%
DEDC-TENG (PI)	427.4	8485	97.6%	95.3%	92.9%	90.5%

Supplementary Table 4. The detailed parameters of different rotation mode DC-TENGs.

Device \ Parameters	Rotation Speed (rpm)	Frequency (Hz)	Area (cm^2)	Power density ($W\ m^{-2}\ Hz^{-1}$)
Ref. 26	600	10	314	0.01
Ref. 18	200	3.3	22	0.02
Ref. 32	300	5	38	0.04
Ref. 27	300	5	15	0.15
Ref. 16	60	1	47	0.30
This work	60	1	25	2.3

Supplementary Table 5. The Coulombic efficiency of microstructure-designed DC-TENG (K = 100) and DC-TENG with insulator (K = 9)

	η(100V)	η(200V)	η(300V)	η(400V)	η(1000V)	η(2000V)
Microstructure-designed DC-TENG	80.1%	60.3%	40.4%	20.6%	_	_
This work	99.2%	98.3%	97.5%	96.6%	91.6%	83.2%

Supplementary Table 6. The Coulombic efficiency of AC-TENG with different structure parameters

	Q_{SC}(nC)	V_{OC}(V)	η(500V)	η(1000V)	η(1500V)	η(2000V)
w/o insulator (0.5 mm)	91.3	638	38.6%	_	_	_
w/o insulator (3.0 mm)	49.4	1704	81.4%	62.7%	44.0%	25.4%
w/ insulator (3.0 mm)	118.1	1806	86.4%	72.8%	59.2%	45.6%

Supplementary Table 7. The Coulombic efficiency of AC-TENG of double dielectric layer

	Q_{SC}(nC)	V_{OC}(V)	η(500V)	η(1000V)	η(2000V)	η(5000V)
w/o insulator	201.2	1193	74.3%	48.6%	_	_
w/ insulator	630.2	5567	96.8%	93.7%	87.4%	68.4%

Supplementary Table 8. The detailed sliding motion parameters

Parameters / Device	Acceleration	Deceleration	Maximum rate	Frequency	Distance	Pressure
DC-TENG (Figure 2)	0.2 m s^{-2}	0.2 m s^{-2}	0.1 m s^{-1}	0.5 Hz	50 mm	10 N
DC-TENG (Figure 2)	0.8 m s^{-2}	0.8 m s^{-2}	0.2 m s^{-1}	1 Hz	50 mm	10 N
CDC-TENG (Figure 4)	0.8 m s^{-2}	0.8 m s^{-2}	0.2 m s^{-1}	1 Hz	50 mm	10 N
DEDC-TENG (Figure 4)	0.2 m s^{-2}	0.2 m s^{-2}	0.1 m s^{-1}	0.5 Hz	50 mm	10 N
AC-TENG (Figure 5)	0.4 m s^{-2}	0.4 m s^{-2}	0.1 m s^{-1}	1 Hz	25 mm	10 N

Supplementary Table 9. The detailed simulation parameters

Permittivity of vacuum (ε_0)	8.854*10^{-12}
Relative permittivity of TL (ε_{r1})	2.2
Relative permittivity of insulator (ε_{r2})	3.5
Surface charge density of TL (μC m^{-2})	-50
Surface charge density of insulator (μC m^{-2})	-50
The electrodes gap of DC-TENG (μm)	100/200/300/400/500/600
The output voltage of DC-TENG (V)	50/100/150/200/250/300

Supplementary Note 1 The charge loss in TENG resulted from power management

The intrinsic output characteristics of TENGs are high output voltage (several kilovolts) and low output current (μA), which are not matched with the input requirement of high input current (mA) and only several volts of normal electronic devices (TENG can only achieve optimal output power when the load voltage is high.). Therefore, the power management circuit (PMC) is indispensable to bridge the gap between the TENG and electronic devices. **Supplementary Fig. 1a** shows a commonly used power management circuit in TENG field. The more energy C_{in} ($E_{in} = 0.5*C_{in}*V_A^2$) stores, the more energy it can be used to drive electronic devices. Therefore, node A has a high potential, which can ensure that TENG generates sufficient output power. Node B has a low potential, which can ensure the normal operation of electronic devices.

The high potential at node A is necessary for efficient power management of TENG. However, with the increase of the potential at node A (It can be controlled by a voltage source in this experiment), the output charge declines rapidly due to a decrease in the surface charge of triboelectric layer caused by electrostatic breakdown (**Supplementary Fig. 2**). In this paper, we report a novel strategy that the spontaneously established reverse electric field (SEREF) between the electrode and triboelectric layer restricts electrostatic breakdown by decreasing the electric field strength below critical breakdown electric field, which can be achieved by only pasting an insulator at the electrode edge (**Supplementary Fig. 2b**).

In addition, the high potential difference between nodes in the power management circuit can also cause electrostatic breakdown, thereby reducing the efficiency of power management circuits. This problem can be solved by encapsulating the power management module, such as circuit encapsulated in a highly insulating epoxy resin to prevent internal breakdown and leakage of electricity[1].

Supplementary Note 2 The actual limitations of CMEO for quantifying the performance of TENGs

In general, to evaluate and compare the performance of TENGs, the standards for quantifying the performance of TENGs were established by the cycle for maximized energy output[2,3,4] (CMEO). The CMEO's *V-Q* curve describe the relationship of the surface charge density (SCD) of triboelectric layer (σ_0), ideal open-circuit voltage ($V_{OC, ideal}$) and the inherent capacitor of TENG (C_T):

$$V_{OC,\ ideal} = \sigma_0 \times S / C_T \qquad (1)$$

where *S* is the effective area of triboelectric layer, the reciprocal of absolute value of the *V-Q* curve slope is the C_T.

Obviously, *S* and C_T is fixed for a fabricated TENG. Therefore, if the SCD remains unchanged, the CMEO's curve can be used to accurately calculate the output energy of TENG (**Supplementary Fig. 3a**). it is worth nothing that the assumption that SCD remains unchanged must be valid regardless of whether TENG is in a short circuit, open circuit, or the large load condition.

However, this assumption is not valid in most cases[5]. As the load of TENG increases gradually, SCD declines gradually, resulting in the actual energy output is lower than the ideal energy output calculated by the CMEO's curve (**Supplementary Fig. 3b**). Besides, the $V_{OC,\ ideal}$ calculated by the CMEO's curve also cannot be obtained in experiment. Because the maximum voltage of TENG is generally determined by the electrode structure, the gap between the electrodes, and the atmosphere in which the electrodes are located.

Based on the above reasons, we think that the standards for quantifying the performance of TENGs based on CMEO are the ideal conditions, which are not suitable for correctly evaluating its practical performance.

Supplementary Note 3 AC-TENG and DC-TENG

According to the differences of working principles, TENGs can be divided into two categories: alternate-current TENG (AC-TENG) based on triboelectrification and electrostatic induction[6], and direct-current TENG (DC-TENG) based on triboelectrification and electrostatic breakdown[7].

The basic working modes of AC-TENGs can be divided into four modes: contact-separation mode, lateral sliding mode, single electrode mode and freestanding triboelectric-layer mode. The working mode of AC-TENG selected in this article is freestanding triboelectric-layer mode due to its high surface charge density and high output energy density.

Supplementary Note 4 The relationship between the three breakdown domains in DC-TENG

According to the physical modeling of DC-TENG, there are three breakdown domains: the first breakdown domain (1st BD) between TL and CCE, the second breakdown domain (2nd BD) between FE and TL, and the third breakdown domain (3rd BD) between CCE and FE **(Fig. 2a)**. Based on the principle of DC-TENG, the electrostatic breakdown in 1st BD is beneficial for the performance of DC-TENG. But, when electrostatic breakdown occurs at 2nd BD, the partial positive charges of FE will directly return to TL, which will lead to a decrease in the performance of DC-TENG. Moreover, with the output voltage ($V_{\text{CCE-FE}}$, the potential difference of CCE and FE) increases **(Fig. 2i)** or the gap between CCE and FE increases **(Supplementary Fig. 17)**, the electrostatic breakdown in 2nd BD is enhanced. The electrostatic breakdown in 3rd BD only occurs when $V_{\text{CCE-FE}}$ is equal to V_{OC}.

Supplementary Note 5 Definition and testing method for the open-circuit voltage of TENG

Ideally, the open-circuit voltage (V_{OC}) of TENG can be calculated by the formula 2.

$$V_{OC,\ ideal} = Q_{SC}/C_T \qquad (2)$$

where C_T is the inherent capacitor of TENG (**Supplementary Note 2**).

In actual conditions, the theoretical V_{OC} generally cannot be obtained, which is mainly due to the unique working mechanism of TENG. On one hand, comparing with the large internal resistance and high voltage characteristics of TENG, the measurement instruments often fail to meet the requirements for the open-circuit condition. On the other hand, the unavoidable parasitic capacitance, the charge loss caused by electrostatic breakdown, and even the structural parameters will influence the achievable V_{OC} in experiment, leading to the measured voltage significantly smaller than the theoretical V_{OC}, which have been demonstrated in many previous works[5, 8]. Therefore, the achievable V_{OC} should be defined as the terminal voltage in the open-circuit state. Obviously, there is no charge transfer in external circuit in this state.

The test circuit in **Fig. 2g** is used to accurately test the output voltage of DC-TENG, where the DC-TENG is in series with the test capacitor (C_{test}), protective resistors (R_{p1} and R_{p2}) and the electrometer to form a closed circuit. The electrometer is used to measure the quantity of flowed charges in the loop. Here, the output voltage of DC-TENG ($V_{DC\text{-}TENG}$) can be regarded as:

$$V_{DC\text{-}TENG}=V_C+V_R \qquad (3)$$

Where V_C is the voltage of capacitor, V_R is the voltage of R_{p1}.

Given that the output current is very small (~1 μA), the V_R is much smaller than V_C. V_R can be neglected, so the following equation can be obtained.

$$V_{DC\text{-}TENG}\approx V_C=\frac{Q}{C_{test}} \qquad (4)$$

where Q is the charge stored in capacitor. Therefore, we can obtain the output voltage of DC-TENG. It is noted that if there is leakage current in the test circuit, the tested charge will be larger than the actual stored charge in the capacitor, and the voltage calculated by equation (3) will be higher than the actual voltage, resulting in inaccurate

voltage measurement. To avoid such inaccurate tests, we also measured the released charge from the C_{test} with the S_1 off and S_2 on (**Fig. 2g<ii>**), and the discharge curve can be regressed to zero, which indicates that the tested charge is equal to the actual stored charge in C_{test}. Finally, in order to eliminate the possibility of the influence of the testing circuit on the experiment results, we also analysis the influence of different testing capacitors on the experiment results (**Supplementary Fig. 12**). In other words, this test method is suitable for measuring the output voltage of DC-TENG.

Supplementary Note 6 The testing method for output energy of TENG

The output energy of TENG for each motion cycle is not affected by the motion frequency. Here, the testing method for output voltage and *Q*-*V* curve of TENG proposed in this work is shown in **Figure 2g**, which can also be utilized to measure the output energy of other TENGs (The schematic diagram of the testing circuit and the process of data processing are shown in **Supplementary Fig. 18**.). The output energy of TENG can be calculated based on the formula ($E_T = 0.5\times C_{test}\times(V_n^2 - V_{n-1}^2)$) (**Supplementary Fig. 18b-d** and **Supplementary Fig. 18f-g**). E_T is the energy generated by TENG in each motion cycle, and V_n is the voltage of C_{test} (the testing capacitor) after the nth motion cycle. It is worth noting that when the voltage of C_{test} reaches the breakdown threshold voltage of the 3rd BD, discharge will occur and the energy stored in C_{test} will stop to increase (**Supplementary Fig. 18e** and **Figure 2j-k**).

Supplementary Note 7 Comparison output power and output energy of TENG

Supplementary Fig. 19 shows the output energy of the six devices proposed in **Figure 3a**. For the devices with the power inflection point (the device 1#, 2#, 3#), the highest output energy will be obtained at the intermediate motion cycle. For devices without the power inflection point (the device 4#, 5#, 6#), the energy in the last cycle is often the highest. The above experimental results indicate that there is a certain similarity between the maximum output power of TENG measured by varying resistances and the maximum output energy measured by capacitances. We consider that the key point is not which method is more suitable for characterizing TENG's performance, but rather the specific conditions under which TENG can achieve its maximum output power/energy.

According to **Supplementary Note 10**, it can be inferred that, if the $\eta(V_{OC}) \leq 50\%$, TENG's output energy is maximum when the output voltage is $V_{\eta = 50\%}$; if the $\eta(V_{OC}) \leq 50\%$, TENG's output energy is maximum when the output voltage is close to V_{OC}. As shown in **Supplementary Fig. 25** and **Supplementary Table 1**, the $\eta(V_{OC})$ of the device 1#, device 2# and device 3# in **Figure 3a** is less than 50%, so there is a power/energy inflection point for these devices. The $\eta(V_{OC})$ of the device 4#, device 5# and device 6# in **Figure 3a** is greater than 50%, so the higher the output voltage, the greater the output power or energy for these devices.

In summary, the Coulombic efficiency we improved in this manuscript indicates that both power density and energy density are suitable for characterizing TENG's performance. For devices with maximum output power inflection point, there is also a maximum output energy inflection point. The inflection point depends on the specific conditions under which TENG can achieve its maximum output power/energy. In comparison, testing the output energy of TENG can provide a more intuitive understanding of the relationship between TENG's output voltage and output energy.

Supplementary Note 8 The maximum output energy of TENG according to CMEO

Ideally, the output energy when the output voltage is V can be obtained by multiplying the horizontal and vertical coordinates of the intersection of the voltage curve and the CMEO's V-Q curve[2]. Therefore, the output energy can be calculated as:

$$E(V) = V\times(Q_{SC}-C_T\times V) \quad (5)$$

as shown in **Supplementary Fig. 20** (the dashed line). When the output voltage is equal to 0.5× $V_{OC,\ ideal}$, the maximum output energy can be calculated as:

$$E_{max} = 0.25\times V_{OC,\ ideal}\times Q_{SC} = 0.25\times Q_{SC}^2\times C_T \quad (6)$$

Therefore, the output energy of TENG is often considered to be proportional to the square of the charge density, or to the product of the short-circuit charge and the open circuit voltage.

Supplementary Note 9 The CREO of TENG

The surface charge density (SCD) of triboelectric layer decreases due to electrostatic breakdown, which cause a decrease in output charge. According to the results of finite element simulation (**Supplementary Fig. 21a-b**), there is a linear relationship between the output voltage (V) and the electric field strength (E), and there is also a linear relationship between surface charge density (σ) and electric field strength. Therefore,

$$E = m_1 \times \sigma + m_2 \times V \qquad (7)$$

Where m_1 and m_2 are constants that are related to the device structure. The threshold electric field for air breakdown is a fixed value (E_{B1}). Therefore, the critical value of the SCD (The corresponding output voltage is 0.) is E_{B1}/m_1.

If the initial surface charge density (The output voltage is 0.) of TL (σ_0) is equal to E_{B1}/m_1. Considering the existence of electrostatic breakdown, the increase of output voltage will enhance the electric field strength around the electrode edge, leading to electrostatic breakdown, and then the SCD of triboelectric layer will decrease. This means that the V-Q curve drawn by formula (1) is not static, and it is a dynamic curve with the increase of TENG's output voltage. We assume that, when the output voltage increases to V_1, the SCD of triboelectric layer will decrease to σ_1. It is noteworthy that the C_T is not affected by electrostatic breakdown. Therefore, substitute σ_1 into formula (1), a new V-Q curve is obtained (the green dashed line in **Fig. 3e**), and the intersection of the voltage curve ($V = V_1$) and the new V-Q curve shifts left. Obviously, the actual output charge (Q'_1) will be lower than the ideal output charge (Q_1) (**Fig. 3e**). When the output voltage further increases to V_2 and V_3, SCD will decrease to σ_2 and σ_3 respectively and the intersection shift left continuously. In addition, when the output voltage increases to V_4, and V_4 equal to $\sigma_4 \times S/C_T$, the output charge will decrease to zero. By connecting these intersections, a new curve (the straight line) for real energy output (CREO) can be obtained, and the product of the horizontal and vertical coordinates of each point on the curve represents the actual output energy of TENG (**Fig. 3e**).

If the initial surface charge density (The output voltage is 0.) of TL (σ_0) is less than E_{B1}/m_1, the electrostatic breakdown does not occur during the initial stage of voltage increase. That means that CMEO can be used to calculated the energy output

(**Supplementary Fig. 21c**). When the output voltage increases to V_1, the electric field reaches to E_{B1}.

$$E_{B1} = m_1 \times \sigma_0 + m_2 \times V_1 \quad (8)$$

This means that a linear increase in output voltage will lead to a linear decrease in surface charge density. When the output voltage further increases to V_2 and V_3, SCD will decrease to σ_1 and σ_2 respectively and the intersection shift left continuously. By connecting these intersections, a new curve (the fold line) for real energy output (CREO) can be obtained (**Supplementary Fig. 21d**).

Supplementary Note 10 The maximum output energy of TENG calculated by *Q-V* curve

Coulomb efficiency of TENG is the ratio of output charge/current to short-circuit output charge/current, which can correctly describe the output energy/power of TENG.

$$\eta(V) = Q(V)/Q_{SC} \quad (9)$$

$$\eta(V) = I(V)/I_{SC} \quad (10)$$

$$E(V) = Q(V)\times V = \eta(V)\times Q_{SC}\times V \quad (11)$$

$$P(V) = I(V)\times V = \eta(V)\times I_{SC}\times V \quad (12)$$

Where $Q(V)$, V, $\eta(V)$, Q_{SC}, $I(V)$, and I_{SC} are the output charge, output voltage, Coulomb efficiency, short-circuit output charge, output current and short-circuit current, respectively. Here, two factors that affects the coulomb efficiency (inherent capacitor of TENG and electrostatic breakdown) are analyzed:

1. There is a capacitor (C_T) in TENG's physical model, which is related to the relative position of electrodes and the size of the electrodes. Therefore, TENG requires some charge (Q_C) to maintain output voltage.

$$V = Q_C/C_T \quad (13)$$

In addition, when TENG is connected to a load, the parasitic capacitance (C_P) in parallel with C_T will cause TENG to require more charge to maintain output voltage.

$$Q_C = V\times(C_T+C_P) \quad (14)$$

where C_T and C_P are constants.

2. The charge density of triboelectric layer can be released by the air breakdown, which causes a decrease in output charge. According to the formula (7) the critical value of the SCD (The corresponding output voltage is 0.) is E_{B1}/m_1. As the output voltage increases, the charge density decreases. Therefore, at a certain voltage, the dissipated charge (Q_B) due to air breakdown can be regarded as:

$$\Delta\sigma = Q_B/S= V\times(m_2/ m_1) \quad (15)$$

where, $\Delta\sigma$ is the variation in surface charge density. Consequently, the final output charge of TENG can be regarded as:

$$Q(V) = Q_{SC} - Q_C - Q_B = Q_{SC} - V\times(C_T+C_P) - V\times(m_2/ m_1) \quad (16)$$

There is a linear relationship between output charge and output voltage:

$$Q(V) = (-a \times V + 1) \times Q_{SC} \quad a \in [0, 1/V_{OC}] \tag{17}$$

$$\eta(V) = Q(V)/Q_{SC} = -a \times V + 1 \quad a \in [0, 1/V_{OC}] \tag{18}$$

More importantly, even though different TENGs have the same Q_{SC} and V_{OC}, their maximum output energy is different. As shown in **Supplementary Fig. 23**. E_{max} can be calculated as:

$$E_{max} = 50\% \times Q_{SC} \times V_{\eta = 50\%} \ (\eta(V_{OC}) \leq 50\%) \tag{19}$$

$$E_{max} = \eta_{V_{OC}} \times Q_{SC} \times V_{OC} \ (\eta(V_{OC}) \geq 50\%) \tag{20}$$

Supplementary Note 11 The Coulomb efficiency of MDC-TENG

To obtain high output current, the integration design of micro-electrode DC-TENG (MDC-TENG) is effective[9]. However, although a small gap between CCE and FE in MDC-TENG can achieve high integration degree and high charge output (charge density increased 40 times), it also leads to low V_{OC} and $\eta(V)$ (S**upplementary Fig. 32a** and **Supplementary Table 5**), thus the power density was only increased by 4 times compared to the initial work about DC-TENG (**Fig. 4k**).

In this work, although K is only 9, and charge density is lower than MDC-TENG, the power density is up to 2.32 W m^{-2} Hz^{-1} due to high Coulomb efficiency **(Fig. 4k)**, which is 54-fold of the previous DC-TENG and 16-fold of the integration design of MDC-TENG. This indicated that the strategy of improving TENG performance solely by increasing charge density has significant limitations.

Supplementary Note 12 Breakdown theory

The breakdown mechanism of gas is usually based on Townsend avalanche, which is described by the empirical relationship between breakdown voltage (V_b) and the product of gap distance (d) and gas pressure (p). Generally, the breakdown criterion is derived based on two important parameters: the electron impact ionization coefficient α and the secondary electron emission coefficient γ.

$$\gamma(e^{\alpha d} - 1) = 1 \quad (21)$$

where α describes the generation of ions by electron impact and the corresponding equation is as follows.

$$\alpha = Ape^{-Bpd/V_b} \quad (22)$$

where A, B are two constants relating to gas compositions, and p is the gas pressure. Thus, the following equation can be obtained.

$$V_b = \frac{Bpd}{\ln(Apd) + \ln\left[\frac{1}{\ln\left(\frac{1}{\gamma}+1\right)}\right]} \quad (23)$$

This is the Paschen curve, and the Paschen's constants for air is: p, 101.25 kPa; A, 10.95 (m×Pa)$^{-1}$; B, 273.8 V (m×Pa)$^{-1}$; γ, 8.136×10^{-3}.

Given that the electric field at the air gap of contact-separation TENG (CS-TENG) is approximately considered as a uniform field, Paschen curve can be used to determine whether air breakdown occurs in CS-TENG, and can also be used to calculate the maximum surface charge density of CS-TENG **(Supplementary Fig. 34)**.

For the sliding mode TENG, the Paschen's law cannot be directly used for calculation, because of the non-ideal conditions. For example, the electric field around the sliding mode TENG is not uniform as assumed in Paschen's law, which depends on the mechanical configuration, triboelectric materials, surface roughness, and other issues. Irrespective of the specific circumstance, there always exists a fixed breakdown threshold for the device that is fabricated (The breakdown could occur at the gap between the electrodes, the gap between the electrodes and the triboelectric layer, etc.).

Besides the demonstration methods in the original manuscript, we also provide another method to demonstrate the mechanism of discharge of sliding mode TENG. The device used in this experiment is the general sliding mode alternating current

TENG (AC-TENG) (**Supplementary Fig. 35a**). The two bottom electrodes remain fixed, and the triboelectric layer are PVDF (polyvinylidene difluoride), PI (polyimide), ETFE (ethylene-terafluoroethlene), PTFE (Polytetrafluoroethylene), PVC (polyvinyl chloride) and FEP (fluorinated ethylene propylene) , respectively. Firstly, we used the strategy proposed in this work to suppress side-discharge of electrodes **Supplementary Fig. 35b <i>**) (Here, the insulating material is the same as the material of triboelectric layer to avoid triboelectrification between these two materials as much as possible.), and measured the surface charge density of triboelectric layer (the purple points in **Supplementary Fig. 35c**). Then, we removed the insulating material between the two bottom electrodes (**Supplementary Fig. 35b <ii>**), and measured the surface charge density of triboelectric layer again (the orange points in **Supplementary Fig. 35c**). It is obviously that the surface charge density of triboelectric layer decays to a very low fixed value regardless of the triboelectric materials. These results demonstrate that the breakdown threshold is fixed when the device structure is determined again.

Supplementary Note 13 Modulating the electric field intensity in the breakdown domain

The strategy proposed in this work for suppressing air breakdown involves introducing charges on the side surface of the insulator and utilizing the electric field established by these charges to modulate the electric field intensity in the air domain (also referred to as the second breakdown domain (2rd BD) of the DC-TENG in the manuscript). It is worth noting that these charges spontaneously accumulate on the side surface of the insulator through air breakdown. This method for modulating the electric field intensity in the breakdown domain has not been reported in the TENG research field.

Besides, we speculate that apart from utilizing surface charge to regulate the electric field, adjusting the terminal voltage of electrode is also a viable method. Here, we introduced an additional metal electrode B at the edge of the existing electrode (referred to as electrode 1 (E1)). Through simulation calculations, we found that the electric field strength at the breakdown region gradually decreases **(Supplementary Fig. 36)** as the voltage applied to electrode 2 (E2) increases. This provides us another approach to suppress air breakdown by modulating the electric field strength. However, as a device for energy harvesting, using an additional voltage source to adjust the voltage applied to electrode B would be redundant for TENG.

Reference

1. He, W. C. et al. Large harvested energy by self-excited liquid suspension triboelectric nanogenerator with optimized charge transportation behavior. *Adv. Mater.* **35**, 2209657 (2023).
2. Zi, Y. L., Niu, S. M., Wang, J., Wen, Z., Tang, W. & Wang, Z. L. Standards and figure-of-merits for quantifying the performance of triboelectric nanogenerators. *Nat. Commun.* **6**, 8376 (2015).
3. Zi, Y. L. et al. Effective energy storage from a triboelectric nanogenerator. *Nat. Commun.* **7**, 10987 (2016).
4. Xia, X., Fu, J. J. & Zi, Y. L. A universal standardized method for output capability assessment of nanogenerators. *Nat. Commun.* **10**, 4428 (2019).
5. Gao, Y. K. *et al.* Achieving high-efficiency triboelectric nanogenerators by suppressing the electrostatic breakdown effect. *Energy Environ. Sci.* **16**, 2304-2315 (2023).
6. Liu, D., Gao, Y. K., Zhou, L. L., Wang, J. & Wang, Z. L. Recent advances in high-performance triboelectric nanogenerators. *Nano Res.* **16**, 11698-11717 (2023).
7. Liu, D. et al. A constant current triboelectric nanogenerator arising from electrostatic breakdown. *Sci. Adv.* **5**, eaav6437 (2019).
8. Liu, J. Q. et al. Achieving ultra-high voltage (≈10kV) triboelectric nanogenerators. *Adv. Energy Mater.* **13**, 2300410 (2023).
9. Zhao, Z. H. et al. Rationally patterned electrode of direct-current triboelectric nanogenerators for ultrahigh effective surface charge density. *Nat. Commun.* **11**, 6186 (2020).
